# Hotspots and frontier trends of diabetic associated cognitive decline research based on rat and mouse models from 2012 to 2021: A bibliometric study

**DOI:** 10.3389/fneur.2022.1073224

**Published:** 2022-12-13

**Authors:** Jie Li, Zhen Wang, Xinyu Nan, Mingjie Yin, Hui Fang

**Affiliations:** ^1^Graduate School of Hebei Medical University, Shijiazhuang, China; ^2^Department of Endocrinology, Tangshan Workers' Hospital, Tangshan, China; ^3^Department of Orthopedics, Handan First Hospital, Handan, China

**Keywords:** diabetic associated cognitive decline, models, bibliometric, cognitive impairment of type 1 diabetes, autophagy

## Abstract

**Background:**

The establishment of rodent models, such as rat and mouse models, plays a critical role in the study of diabetic associated cognitive decline. With the continuous growth of relevant literature information, it is difficult for researchers to accurately and timely capture the topics in this field. Therefore, this study aims to explore the current status and frontier trends of diabetic associated cognitive decline research based on rat and mouse models through a bibliometric analysis.

**Methods:**

We collected 701 original articles on this subject from the Science Citation Index Expanded of the Web of Science Core Collection from 2012 to 2021. Then we utilized CiteSpace and VOSviewer for plotting knowledge maps and evaluating hotpots and trends.

**Results:**

During this decade, except for a slight decline in 2020, the number of annual outputs on diabetes associated cognitive decline research using rat and mouse models increased every year. China (country), China Pharmaceutical University (institution), Gao, Hongchang (the author from the School of Pharmacy of Wenzhou Medical University, China), and Metabolic Brain Disease (journal) published the most papers in this research field. The analysis results of co-cited references and co-occurrence keywords indicated that “mechanisms and prevention and treatment methods”, especially “oxidative stress”, “potential association with Alzheimer's disease” and “spatial memory” are research focuses in this subject area. The bursts detection of references and keywords implied that “cognitive impairment of type 1 diabetes” and “autophagy and diabetes associated cognitive decline” will be potential directions for future research in this subject area.

**Conclusion:**

This study systematically assessed general information, current status and emerging trends of diabetic associated cognitive decline research using rat and mouse models in the past decade based on a bibliometric analysis. The number of publications was annually increasing although a slight decline was observed in 2020. Contributions from different countries/regions, institutions, authors, co-cited authors, journals and co-cited journals were evaluated, which may also be used to guide future research. Through the analysis of references and keywords, we predicted the future research hotspots and trends in this field.

## Introduction

According to the study of the International Diabetes Federation (IDF), the number of patients with diabetes mellitus (DM) will rise to 628 million by 2045. And with the annual growth of patients with DM, the incidence of diabetic complications is also on the rise (1). DM is a metabolic disease related to the increased risk of central nervous system diseases (2), which can lead to electrophysiological and structural changes in the nervous system, thus resulting in cognitive decline and even dementia (3, 4). A retrospective study showed that DM increased the risk of cognitive impairment and various types of dementia by 1.25–1.91 times (5). In recent years, with the increase of researchers' interests in diabetes associated cognitive decline (DACD), more and more research articles have been published, involving the pathogenesis, evaluation means, prevention and treatment methods. In these medical research experiments, the establishment of rodent models, such as rats and mice, is of great value for understanding the pathogenesis and development of new drugs of DACD. However, with the continuous growth of literature information, it is difficult for researchers to accurately and timely capture the topics in this field. Therefore, for a large number of DACD studies based on rat and mouse models in recent years, it is necessary to conduct a bibliometric analysis on this subject to present the face of this research field.

Pritchard first proposed bibliometric analysis in 1969, which is a science and technology using statistical and mathematical methods to identify key articles and topics. CiteSpace is a bibliometric visualization software developed by Professor Chen Chaomei and others using Java language (6, 7). VOSviewer is a bibliometric analysis tool developed by Nees Jan van Eck and Ludo Waltman of Leiden University in the Netherlands in 2009 (8). These two software are used to analyze the status and development trends in a certain period by drawing knowledge maps, which have been widely used by medical and biological scholars (6).

In this study, on the basis of CiteSpace and VOSviewer, we systematically and intuitively analyzed the characterizing hotpots and frontier landscapes of DACD research using rat and mouse models in the past decade, which could provide clues for researchers to determine new research directions.

## Materials and methods

### Data source and search strategy

Science Citation Index Expanded (SCI-E) of the Web of Science Core Collection (WoSCC) was the data source in this study. The search strategy was defined as TS (Topic Search) =rat model OR mouse model OR diabetic rat OR diabetic mouse AND diabetic associated cognitive decline OR diabetic cognitive impairment OR diabetic encephalopathy. Topic search includes title, summary, author, and keywords. The time frame was from 2012-01-01 to 2021-12-31, and the language was limited to English. Further, the article type was chosen as “Article”. And reviews, conference papers, book chapters and withdrawn publications were excluded. A total of 1,397 literature records had been obtained. These complete records and references, including title, authors, abstract and references, were exported in plain text format. Two authors screened out articles that do not conform to the subject by reading the title, abstract or full text. Then the data was imported into CiteSpace, and 701 documents were obtained after removing duplication.

### Data analysis

First, Microsoft Office Excel 2019 was used to represent the trend of the number of articles published each year.

Then, CiteSpace (6.1 R3) was used to conduct visual network analysis on 701 articles. The specific parameters were set as follows: time slice (January 2012–December 2021), slice year (1), source of terms (title, abstract, author keywords and keywords), link strength (cosine), selection criteria (TOP *N* = 50), pruning (None). Country, institution, keyword and reference were selected as the node type for analysis respectively. In the generated maps, nodes represent countries, institutions, keywords, and references respectively. The size of a node represents the number of occurrences. The link represents the cooperation, co-occurrence or co-cited between two nodes. The color of a node and link represents the release or cited time. From 2012 to 2021, the color changes gradually over time. The purple circle of the node means that it has a high centrality (≥0.10). The greater the centrality of a node, the more influence it has on other nodes. In addition, we conducted bursts detection of references and keywords, which can help researchers infer research hotpots and frontiers. The blue line in the picture of bursts detection represents the time interval, and the red line represents the duration of the citation bursts.

Finally, VOSviewer (1.6.18) was used to visually analyze the authors and co-cited authors, journals and co-cited journals. In the network visualization, different colors of nodes denote corresponding clusters. The distance between two nodes indicates the closeness and similarity. The size of a node stands for the frequency of occurrence.

## Results

### Trends in annual publications

**Figure 1:**
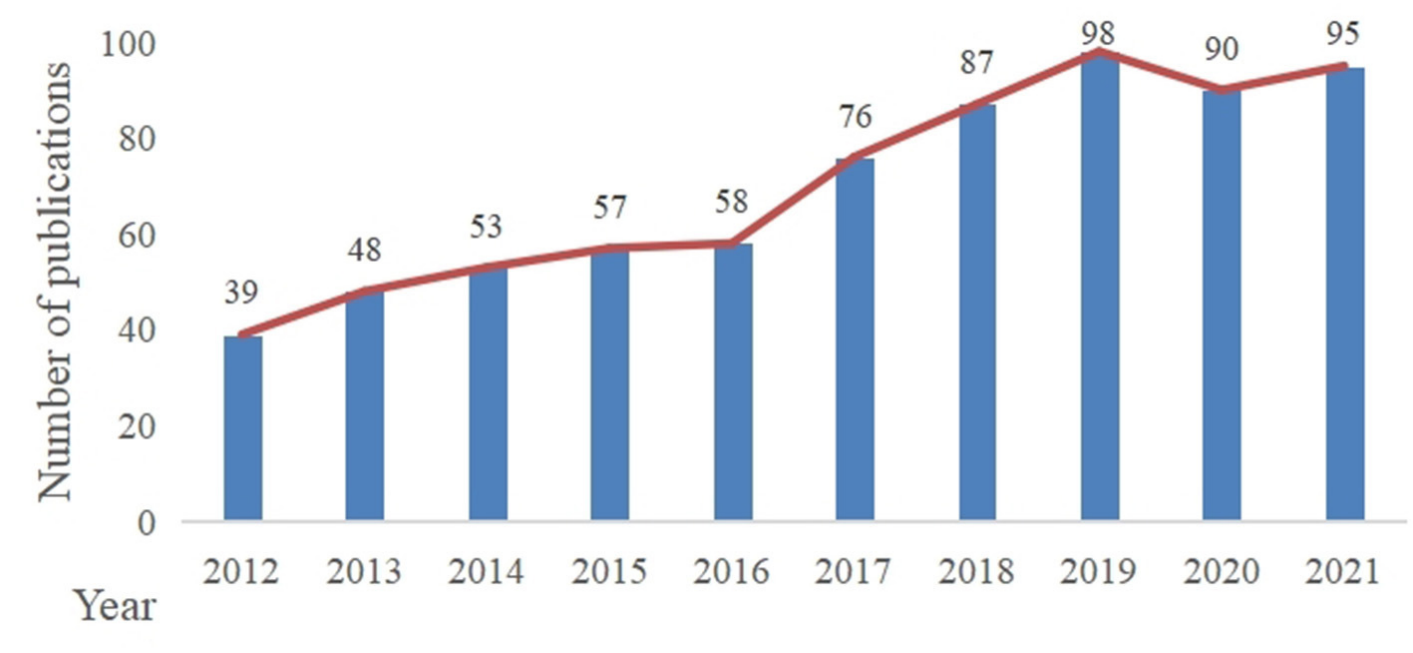
The number of annual publications from 2012 to 2021 in this field.

In our study, 701 documents were included in the analysis. As shown in Figure 1, the number of annual global publications on DACD research using rat and mouse models showed a steady growth trend by 2020. After a slight decline in 2020, the number was rise again in 2021.

### Analysis of countries/regions and institutions contribution

**Figure 2:**
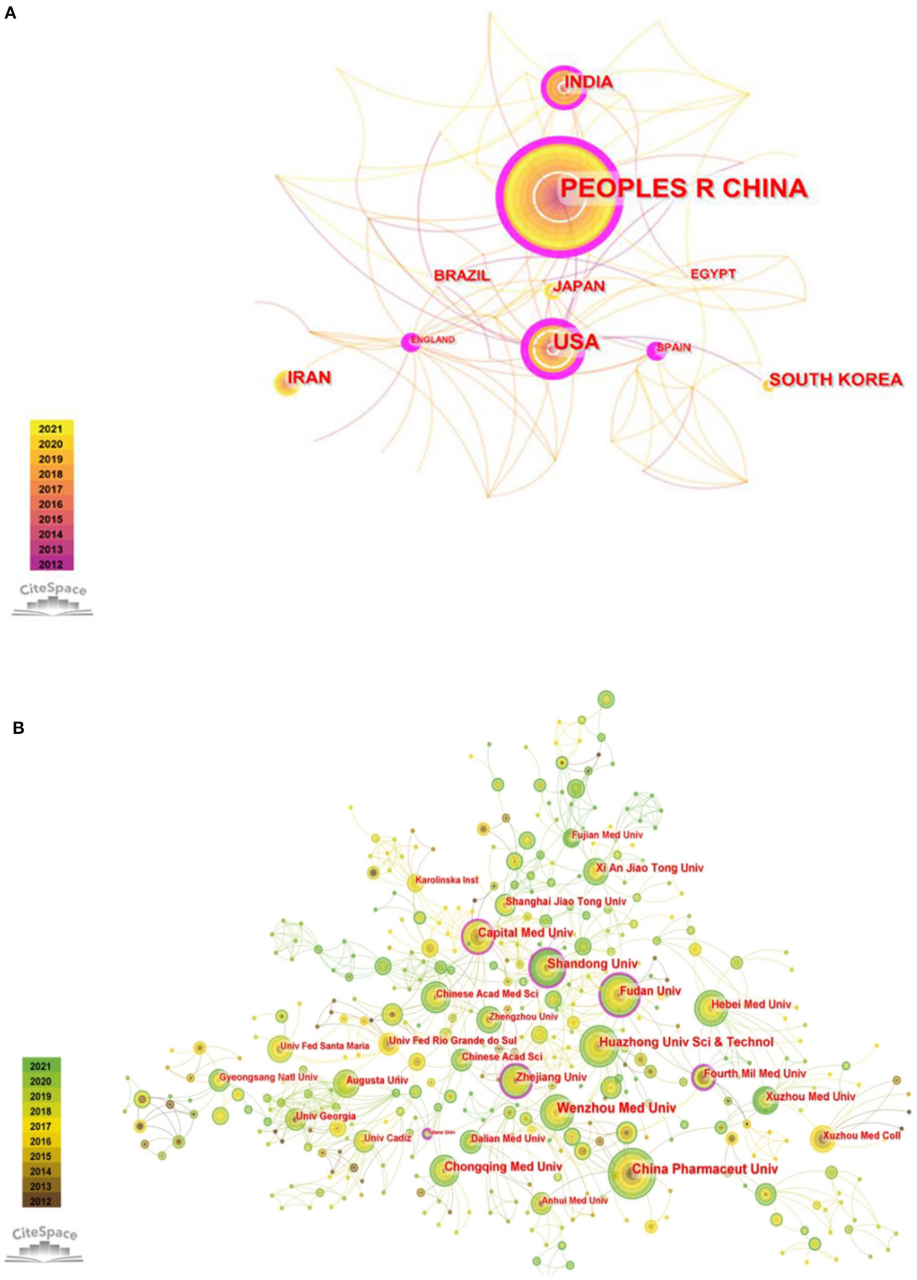
**(A)** The network visualization of countries/regions collaboration. **(B)** The network visualization of institutions collaboration.

From 2012 to 2021, a total of 53 countries/regions made contributions to publishing articles in this field. The collaboration of countries/regions is visualized in Figure 2A. Supplementary Table 1 lists the top 10 countries/regions and institutions with the highest productivity and centrality. China published the most articles (*n* = 351), followed by the United States (*n* = 96) and India (*n* = 47). The top three countries on centrality were the United Kingdom (*n* = 0.43), the United States (*n* = 0.4), and China (*n* = 0.35).

In total, 828 institutions participated in the subject research. As shown in Figure 2B, the institutions that published the most papers were China Pharmaceutical University (*n* = 20), followed by Wenzhou Medical University (*n* = 19) and Shandong University (*n* = 17), which are all from China. In terms of centrality, the top three institutions were Zhejiang University (*n* = 0.17), Tulane University (*n* = 0.15) and Fudan University (*n* = 0.14). The centrality of these three institutions was >0.10, which indicated that they have greater cooperation with other institutions in this research field (Supplementary Table 1).

### Analysis of authors and co-cited authors

**Figure 3:**
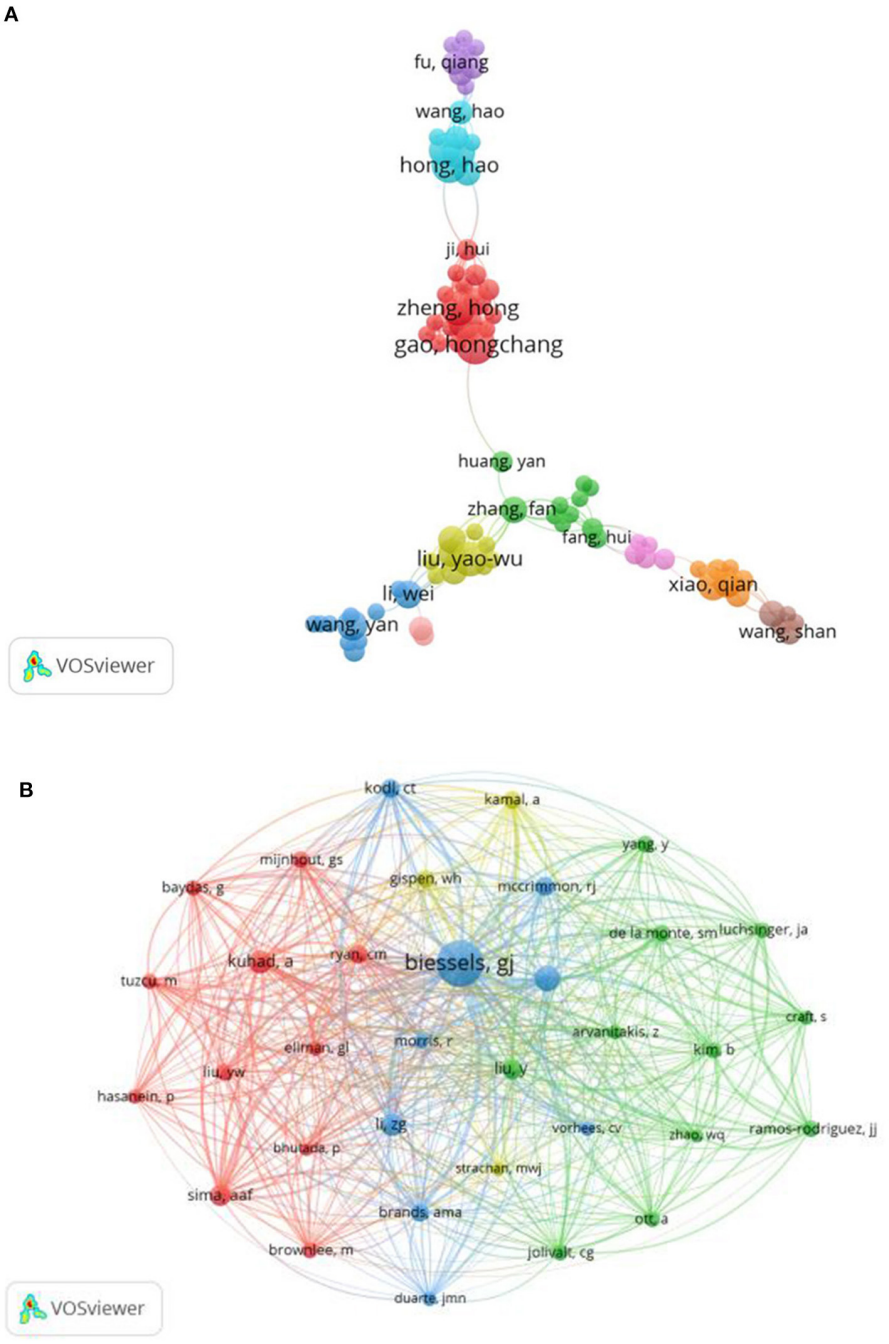
**(A)** The network visualization of authors collaboration. **(B)** The network visualization of co-cited authors with the minimum citations over 40 times.

These publications involved 3,837 authors. The top three authors who produced the highest number of articles were Gao, Hongchang (*n* = 13), Ergul, Adviye (*n* = 11) and Zheng, Hong (*n* = 10) (Figure 3A, Supplementary Table 2). Figure 3B shows the network of co-cited authors. Biessels, GJ was the first cited author with 363 citations, followed by Stranahan, AM (*n* = 118) and Kuhad, A (*n* = 104) (Supplementary Table 2).

### Analysis of journals and co-cited journals

A total of 635 academic journals published articles on DACD research based on rat and mouse models. Table 1 shows the top ten journals in terms of the number of publications and citations on this topic field. During this decade, the number of articles published on Metabolic Brain Disease was the largest (*n* = 25), followed by Brain Research (*n* = 18) and Molecular Neurology (*n* = 14). In addition, the result of this study showed that the top three co-cited journals were Diabetes (*n* = 971), Brain Research (*n* = 742) and Diabetologia (*n* = 617).

**Table 1:** The top 10 journals and co-cited journals.

**Rank**	**Journal**	**Counts**	**IF (2021)**	**JCR (2021)**	**Co-cited journal**	**Counts**	**IF (2021)**	**JCR (2021)**
1	Metabolic Brain Disease	25	3.655	Q3	Diabetes	971	9.337	Q1
2	Brain Research	18	3.610	Q3	Brain Research	742	3.610	Q3
3	Molecular Neurobiology	14	5.682	Q1	Diabetologia	617	10.460	Q1
4	Neuroscience	14	3.708	Q3	Journal of Neuroscience	583	6.709	Q1
5	Neuroscience Letters	13	3.197	Q3	European Journal of Pharmacology	581	5.195	Q2
6	Plos One	13	3.752	Q2	Plos One	559	3.75	Q2
7	Behavioural Brain Research	12	3.352	Q2	Diabetes Care	533	17.152	Q1
8	Neurochemica Research	12	4.414	Q2	Journal of Biological Chemistry	533	5.486	Q2
9	Brain Research Bulletin	11	3.715	Q3	Proceedings of the National Academy of Sciences of the United States of America	529	212.779	Q1
10	Aging-Us	10	5.955	Q2	Journal of Alzheimer's Disease	515	4.610	Q2

IF, impact factor; JCR, journal citation reports.

### Analysis of co-cited references and references bursts

In the process of analyzing references by CiteSpace, when the select criteria was TOP *N* = 50, the nodes would be prompted to exceed the limit and cannot be displayed. Therefore, the select criteria in references analysis was TOP *N* = 30. The most frequently co-cited reference can reflect the research achievements most concerned by researchers in relevant research fields. As shown in Table 2, the reference with the highest number of citations was a review on the pathogenic mechanism and clinical significance of DCAD, which was written by Biessels, GJ in 2018 and published on Nature Reviews Endocrinology (IF = 47.564). From 2012 to 2021, the number of citations in this subject research reached 25 times. The second most cited article was “Effects of metformin on inflammation and short-term memory in streptozotocin-induced diabetic mice” published in Brain Research (*n* = 19). “Diabetes and Cognitive Impairment” published in Current Diabetes Reports was the third most cited reference (*n* = 16).

**Table 2:** The top 10 co-cited references.

**Rank**	**Title**	**Cited frequency**	**First author**	**Publication year**	**Type**
1	Cognitive decline and dementia in diabetes mellitus: mechanisms and clinical implications	25	Biessels, GJ	2018	Review
2	Effects of metformin on inflammation and short-term memory in streptozotocin-induced diabetic mice	19	Oliveira, WH	2016	Article
3	Diabetes and Cognitive Impairment	16	Zilliox, LA	2016	Review
4	The Effect of Diabetes Mellitus on Apoptosis in Hippocampus: Cellular and Molecular Aspects	15	Sadeghi, A	2016	Review
5	Astrocytic and microglial response in experimentally induced diabetic rat brain	15	Nagayach, A	2014	Article
6	Inside the Diabetic Brain: Role of Different Players Involved in Cognitive Decline	15	Gaspar, JM	2016	Review
7	Encephalopathies: the emerging diabetic complications	14	Sima, AAF	2010	Review
8	Diabetes-accelerated memory dysfunction via cerebrovascular inflammation and Abeta deposition in an Alzheimer mouse model with diabetes	13	Takeda, S	2010	Article
9	Impact of diabetes on cognitive function and brain structure	12	Moheet, A	2015	Review
10	A look inside the diabetic brain: Contributors to diabetes-induced brain aging	12	Wrighten, SA	2009	Review

**Figure 4:**
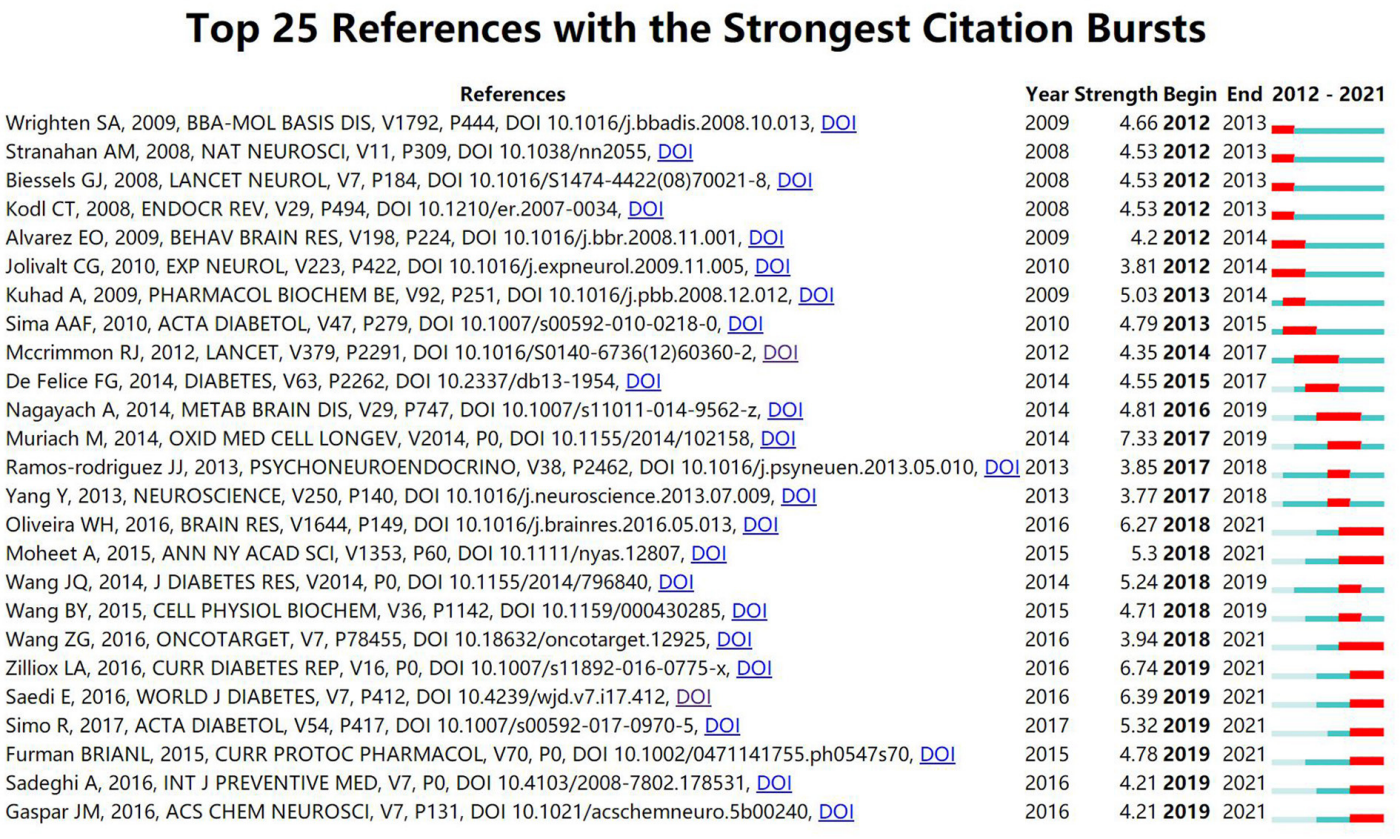
The top 25 references with the strongest citation bursts.

In addition, Figure 4 displays the top 25 references with the strongest citation bursts. The reference “Diabetes and the brain: oxidative stress, inflammation, and autophagy” written by Muriach, M had the highest burst strength (*n* = 7.33). Moreover, Furman BRIANL (2015), Sadeghi, A (2016), and Gaspar, JM (2016) have gained more attention in recent years.

### Analysis of co-occurrence keywords and keywords bursts

**Figure 5:**
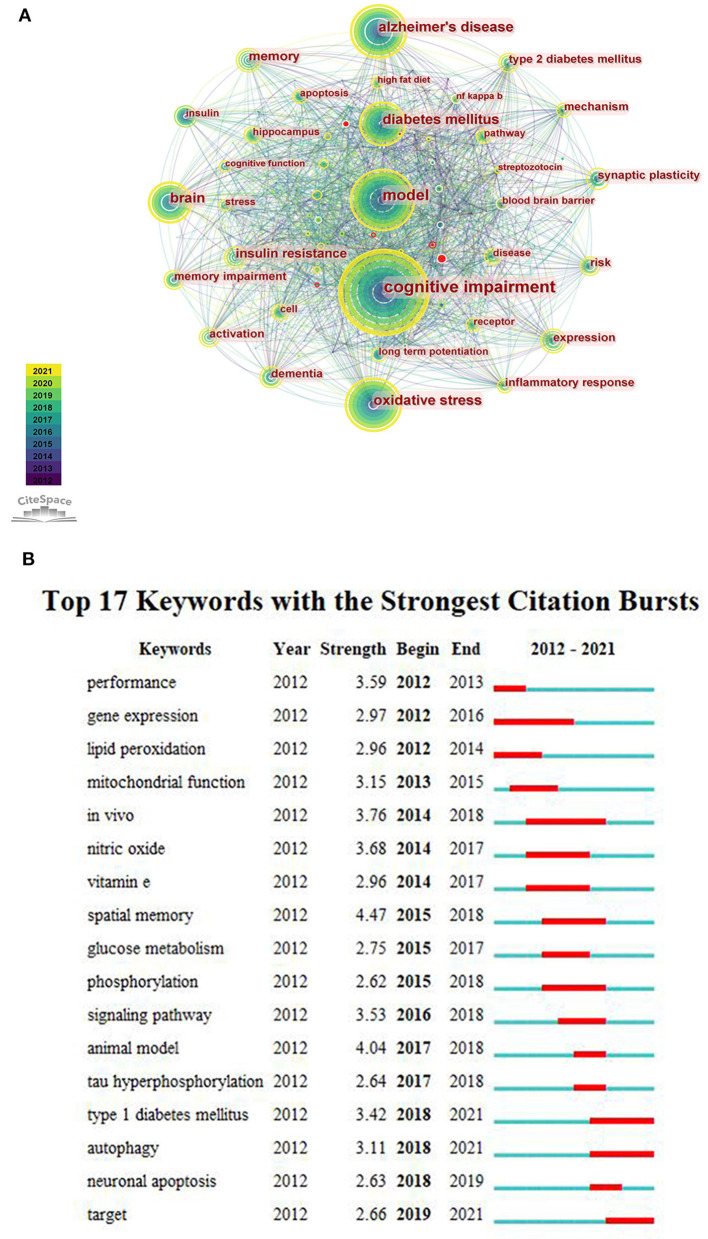
**(A)** The co-occurrence network of keywords. **(B)** The top 17 keywords with the strongest citation bursts.

We merged some keywords with similar meanings. According to the generated network map, the larger nodes were “cognitive impairment”, “Alzheimer's disease”, “model”, “oxidative stress” and “diabetes mellitus” (Figure 5A). It indicated that these keywords appear more frequently, and they were popular topics in this research field. Figure 5B represents that the focus of scholars' have changed over time, and “type 1 diabetes”, “autophagy” and “target” have attracted attention in recent years. In addition, it was found that the burst strength of “spatial memory” was the strongest (*n* = 4.47).

## Discussion

Unlike systematic review and meta-analysis, the bibliometric study focuses on qualitative and quantitative analysis of countries, institutions, authors, journals, references, and keywords in the research literature on a specific topic, so as to systematically and visually assess the previous research hotspots and development trends in this field (9). In this paper, we conducted a bibliometric analysis of 701 original articles from SCI-E of WoSCC from 2012 to 2021 based on DACD research using rat and mouse models.

### General information

From 2012 to 2019, there was a steady increase in outputs in this research theme. After a small reduction in 2020, it rose again in 2021. We speculated that the slight decline in 2020 may be due to the outbreak of the COVID-19 pandemic, which led to the delay in experimental progress and articles production. With the control of the epidemic, the number of articles published began to increase in 2021, which indicated that this field is still a hot topic and has a positive future trend. According to the analysis of countries/regions and institutions, China ranked first in the number of publications, and the top ten institutions in the number of publications were all from China. In addition, China ranked third in centrality. So it implied that China has a certain international influence and a high level of research quality. Although England had not published many articles, it was the leading country with the highest centrality, indicating that it has a close cooperation relationship with other countries/regions. The United States ranked second in the number of publications and centrality, which implied that the United States has a high scientific strength and a leading position in this topic subject. Through the analysis of countries/regions and institutions, it is helpful for researchers to seek cooperation and academic exchanges in the study process.

In the analysis of authors, Gao, Hongchang and Zheng, Hong (the first and third authors in the number of producing papers) are a team and come from the School of Pharmacy of Wenzhou Medical University in China (the institution with the second largest number of published publications). Their research emphasis was to explore the metabolic changes associated with DACD based on rat and mouse models, making great contributions to the research in this subject area (10–13). In the analysis of co-cited authors, Biessels, GJ from the Department of Neurology, Brain Center Rudolf Magnus and University Medical Center Utrecht was the most co-cited author. Meanwhile, “Cognitive decline and dementia in diabetes mellitus: mechanisms and clinical implications” written by Professor Biessels, GJ on Nature Reviews Endocrinology in 2018 was the most co-cited reference (4). This review describes in detail the characteristics, risk factors and pathological manifestations of cognitive impairment in different stages of DM, providing important clues for prognosis and treatment, and had been cited 25 times.

In the journals analysis, Metabolic Brain Disease ranked first in the number of publications published, with 25 articles on the subject published in the past decade. The top ten journals with the largest number of publications are shown in Table 1, and their impact factors are all more than 3, mainly involving endocrinology, neurology, molecular biology and comprehensive medicine, which can help researchers in this field quickly find appropriate journals to submit and publish their new research results. The analysis of co-cited journals could show the contribution of each journal to this field. Of the top 10 co-cited journals, nine are distributed in the Q1/Q2 region, with only Brain Research belonging to Q3 (Table 1), which revealed that high-quality research results attract more attention from researchers in this field.

### Research hotspots

In the analysis of cited references, 7 of the top 10 references were systematic reviews (Table 2), that focus on summarizing the complex pathophysiological mechanism of DACD and providing insights for the prevention and improvement of this complication of DM in the future (4, 14–19). In addition, the strongest bursts citation reference was also a systematic review that elaborating on the mechanism of oxidative stress, autophagy and inflammation in diabetic brain injury (20). The remaining three articles were all original articles on the basis of rodent models. Oliveira et al. (21) used streptozotocin (STZ)-induced diabetic mice to prove that metformin could significantly improve hippocampal neuroinflammatory and neuron loss, thereby promoting the improvement of cognitive and memory function (21). Nagayach et al. (22) confirmed that cell death related to DM leads to abnormal activation of astrocytes and microglia in the hippocampus of rats, causing damage to neurons (22). Takeda et al. (23) analyzed the metabolic and pathologic changes in mice brains by hybridization models between Alzheimer's disease transgenic mice and two types of diabetic mice. They revealed that DM could accelerate memory dysfunction through cerebrovascular inflammation and Aβ deposition (23). In general, these articles well reflect the past and ongoing research directions of DACD and provide good instructions in this field. The above showed that although much progress had been made in understanding DACD, its complex mechanisms and prevention and treatment methods are still the research focus in this topic subject, and researchers need to continue to conduct more intensive studies.

In the co-occurrence keywords analysis, three of the top five terms were the subject words of our article, indicating that these literature are mainly around “cognitive impairment”, “model” and “diabetes mellitus”. The other two were “Alzheimer's disease” and “oxidative stress”. Rodent models are of great importance in DACD research. It is not only help in understanding the pathophysiology of DACD but also in evaluating new therapeutic drugs. The first step to construct DACD rodent models is to build DM rodent models. The common rat and mouse models of DM can be divided into spontaneous models, induced models and transgenic technology models (Table 3) (24–27). Rat and mouse models induced by high-dose STZ injection are widely used in building T1DM models. The use of a high-fat diet to induce insulin resistance, followed by low to moderate doses of STZ to develop mild to moderate insulin deficiency, may currently be the most frequently used method for establishing T2DM models. After DM rodent models are successfully constructed, cognitive function impairment could be evaluated over time through the Morris water maze test, novel object recognition test, inhibitory avoidance test and so on. Compared with other species, rat and mouse models have obvious advantages including animal size, short induction period, easy DM induction, and economic efficiency. However, none of these models precisely mimic human DM, researchers should choose suitable models for a particular experiment based on various factors.

**Table 3:** The methods of common rat and mouse models of DM.

**Diabetes models**	**Type 1 diabetes**	**Type 2 diabetes**
Spontaneous models	Biobreeding rat, LEW 1AR1/-iddm rat, Nonobese diabetic mouse, etc.	Zucker diabetic fatty rat, Goto-Kakizaki rat, KK-AY mouse, Lep ob/ob mouse, Lepr db/db mouse, etc.
Induced models	Repeated low-dose STZ injection. A single high-dose STZ injection. Alloxan injection. Pancreatectomy.	A high-fat diet feed and low to moderate doses of STZ injection. STZ and nicotinamide injection.
Transgenic technology models	NOD/Ltsz-Rag1^null^ mouse, Nude mouse, NOD/LtSz-scid mouse, etc.	MKR mouse, GK/IRS-1double gene deletion mouse, etc.

STZ, streptozocin.

Alzheimer's disease (AD) is a neurodegenerative disease with cognitive impairment as its main clinical manifestation. Previous studies have shown that the incidence of AD in diabetic patients is significantly increased, which inferred that AD is closely related to DM (28, 29). Therefore, more and more researchers used rats and mice as models to explore and confirm the similar pathogenesis between AD and DACD, such as insulin resistance, hyperglycemia, cerebrovascular disease, neuroinflammation, mitochondrial dysfunction, isolated insulin degrading enzymes, etc. (29, 30). Because of the potential relationship between AD and DACD, the researchers also confirmed that the treatment and application of antidiabetic drugs such as glibenclamide, pioglitazone, vengliptin, metformin and so on can effectively control or reverse the progress of AD through rat and mouse models experiments, thus providing new ideas for the diagnosis and treatment of AD (31–34).

Oxidative stress is one of the most important pathogenesis of DM. Under normal conditions, aerobic metabolism will continuously produce reactive oxygen species (ROS). The imbalance of ROS production and clearance in cells or body will result in oxidative stress. Compared with other tissues, the brain has a high oxygen consumption, so mitochondria, as an important organelle for energy production, plays an important role in maintaining brain functional stability and meeting the energy demand of neurons. At the same time, the brain is more vulnerable to oxidative damage because of its relatively low antioxidant defense capacity (35). A number of animal experimental studies have shown that chronic hyperglycemia leads to enhanced oxidative stress by mediating multiple signaling pathways. And these signal pathways also are affected by oxidative stress. It will eventually cause neuronal apoptosis and neuroinflammation, and then cause cognitive dysfunction (15, 20). In addition, the application of antioxidants such as lutein and DHA have been proven to improve the oxidative stress injury induced by DM, which may be beneficial to the central nervous system of diabetic patients (36, 37). Therefore, exploring oxidative stress and its chain reaction has become the focus of research in this subject area, which will help us find an important target to improve the cognitive impairment of diabetes.

Spatial memory is the most explosive keyword for citation bursts. The cognitive dysfunction of DM is mainly manifested as learning-memory impairment and spatial orientation disorder. Therefore, the Morris water maze test is used to test the learning-memory and spatial orientation disorders of diabetic rats in experimental research, so as to explore the pathogenesis or therapeutic targets, which has become one of the research focuses in this subject area in recent years. As we all know, the hippocampus is responsible for receiving information processing, memory and signal transmission, and is an important area closely related to cognitive functions such as learning-memory. Animal experiments showed that the synaptic plasticity in the hippocampus is closely related to cognitive functions related to learning and memory. With the prolongation of the course of DM, the decreased expression of long-term potentiation (LTP) and increased expression of long-term inhibition (LTD) in hippocampus synapses, the decline in the number of synapses, and widen synaptic cleft, can cause changes in synaptic plasticity, thereby effecting spatial learning-memory (38). In addition, some studies confirmed that because of DM, the ultrastructure of nerve cells in the hippocampus is damaged, subsequently some neurons cells apoptosis occurs such as karyopyknosis and cell volume reduction in advance, which may also be one of the reasons for memory hypofunction (15).

### Future research directions

Bursts detection can reflect the emerging trends in the scientific field and the potential direction of future research. Combined with recent years' attention to keywords and references with the strongest citation bursts, this paper summarizes future potential research directions of DACD based on rat and mouse models.

#### Cognitive dysfunction in T1DM

Generally speaking, both T1DM and type 2 diabetes mellitus (T2DM) have different degrees of cognitive dysfunction, but their clinical manifestations are different. Cognitive impairment of T2DM is common in middle-aged and elderly diabetic patients, and is mainly manifested in memory and complex information processing disorders (39). However, the cognitive impairment of patients with T1DM mainly shows the defects of explicit memory, problem solving and intellectual process, which mostly occurs in the patients' adolescence (39, 40). Adolescence is the key period to determine intelligence quotient, and the number of patients with T1DM is increasing, so researchers are paying more and more attention to this group. Previous studies demonstrated that cognitive dysfunction in T1DM is closely related to abnormal blood glucose fluctuations, insulin deficiency, neurotrophic factor deficiency, excessive oxidative stress, calcium homeostasis damage and other factors (41, 42). In addition, abnormal pathomorphological changes such as decreased brain volume, changes in cerebral perfusion, and increased cortical atrophy were found in the STZ-induced T1DM mouse models (43). Although there is a certain understanding of the cognitive impairment caused by T1DM, there are still many uncertainties. In the future, extensive and in-depth research utilizing rat and mouse models is still needed, so as to find new strategies to prevent and treat the cognitive decline of T1DM.

#### Autophagy and DACD

Autophagy is a protective mechanism in cells. Under certain stimulation, cells can wrap internal misfolded proteins, damaged organelles and invading pathogens to form independent membrane structures, and transport them to lysosomes for degradation to form small molecules such as amino acids. More and more evidences identified that autophagy plays a key role in AD-like pathological changes, neuroinflammation, synaptic plasticity and oxidative stress, which are all related to the development of DACD (44). In addition, drug applications targeting autophagy signals, such as some antidiabetic drugs and traditional Chinese medicine, rapamycin, and melatonin, can enhance autophagy and improve learning-memory disorders (44). However, it has been reported that excessive activation of autophagy may lead to aggravation of cerebral ischemia in models of DM with ischemic encephalopathy, whereas the application of autophagy inhibitors can alleviate brain injury (45). Therefore, autophagy may play a dual role in DACD. In the future, researchers need to further verify the specific molecular biological mechanism, and explore the development of autophagy-targeted drugs.

## Limitations and conclusions

As far as we know, this study is the first bibliometric analysis to investigate the current situation and trend of DACD research based on rat and mouse models, but our study has the following limitations. First, in order to meet the format requirements of CiteSpace and VOSviewer, only the documents in SCI-E of WoSCC were included in this study. Other databases such as PubMed, Google Scholar and Scope are not included, so this study may not fully represent the available information in this field. Moreover, we only analyzed the original articles on English manuscripts in this subject area in the past decade. It is necessary to be presented from a wider perspective for this field in the future.

The importance of the establishment of rodent models in the research field of DACD has been recognized. This bibliometric study analyzed the research literature information on DACD using rat and mouse models from 2012 to 2021, and showed the network visualization of countries/regions, institutions, authors and journals. In addition, through the analysis of references and keywords, we evaluated the research hotspots and emerging trends in this subject area. The research hotspots in this field are the mechanisms and treatment of DACD, especially oxidative stress, potential association with Alzheimer's disease and spatial memory. In the future, more studies using rat and mouse models on cognitive impairment of T1DM should be conducted, and the association between autophagy and DACD is worth further studies. In a word, this paper can provide guidance for researchers in this field to seek cooperation and determine research directions.

## Data availability statement

The raw data supporting the conclusions of this article will be made available by the authors, without undue reservation.

## Author contributions

JL and HF designed the study. JL reviewed the literature and drafted the manuscript. ZW, XN, and MY analyzed the data. HF revised the manuscript for final publication and provided funding support. All authors contributed to the article and approved the submitted version.
