# Supplementary material for: Hotspots and frontier trends of diabetic associated cognitive decline research based on rat and mouse models from 2012 to 2021: A bibliometric study
Source: Front Neurol. 2022 Dec 13;13:1073224. doi: 10.3389/fneur.2022.1073224 (PMC9793002; doi:10.3389/fneur.2022.1073224)
Supplement: Supplementary file 1 [file Data_Sheet_1.docx]

Supplementary Material

# Supplementary Tables

**Table 1**  Top 10 countries/regions, institutions in terms of publications and centrality

| Items | Rank | Counts | Name | Rank | Centrality | Name |
| --- | --- | --- | --- | --- | --- | --- |
| Country | 1 | 351 | People’s Republic of China | 1 | 0.43 | England |
|  | 2 | 96 | United States | 2 | 0.4 | United States |
|  | 3 | 47 | Indian | 3 | 0.35 | People’s Republic of China |
|  | 4 | 44 | Iran | 4 | 0.32 | Spain |
|  | 5 | 34 | South Korea | 5 | 0.2 | Indian |
|  | 6 | 30 | Japan | 6 | 0.15 | Saudi Arabia |
|  | 7 | 21 | Brazil | 7 | 0.11 | France |
|  | 8 | 20 | Egypt | 8 | 0.1 | Germany |
|  | 9 | 11 | Turkey | 9 | 0.09 | Denmark |
|  | 10 | 11 | Spain | 10 | 0.08 | Egypt |
| Institution | 1 | 20 | China Pharmaceutical University | 1 | 0.17 | Zhejiang University |
|  | 2 | 19 | Wenzhou Medical University | 2 | 0.15 | Tulane University |
|  | 3 | 17 | Shandong University | 3 | 0.14 | Fudan University |
|  | 4 | 17 | Huazhong University of Science & Technology | 4 | 0.13 | Capital Medical University |
|  | 5 | 16 | Capital Medical University | 5 | 0.11 | Shandong University |
|  | 6 | 16 | Chongqing Medical University | 6 | 0.11 | Fourth Military Medical University |
|  | 7 | 14 | Fudan University | 7 | 0.09 | Massachusetts General Hospital |
|  | 8 | 12 | Zhejiang University | 8 | 0.08 | Harvard Medical School |
|  | 9 | 12 | Xuzhou Medical School | 9 | 0.06 | China Pharmaceutical University |
|  | 10 | 12 | Xi’an Jiao Tong University | 10 | 0.06 | Huazhong University of Science & Technology |

**Table 2**  Top 10 autors in terms of counts

| Rank | Author Name | Counts | Rank | Co-cited author Name | Counts |  |
| --- | --- | --- | --- | --- | --- | --- |
| 1 | Gao Hongchang | 13 | 1 | Biessels, GJ | 363 |  |
| 2 | Ergul Adviye | 11 | 2 | Stranahan, AM | 118 |  |
| 3 | Zheng hong | 10 | 3 | Kuhad, A | 104 |  |
| 4 | Liu Yaowu | 10 | 4 | Li, ZG | 91 |  |
| 5 | Hong Hao | 9 | 5 | Sima, AAF | 82 |  |
| 6 | Long Yan | 9 | 6 | Liu, Y | 74 |  |
| 7 | Zhao Liangcai | 9 | 7 | Kodl, CT | 71 |  |
| 8 | Hu Mei | 8 | 8 | Mccrimmon, RJ | 67 |  |
| 9 | Li Weiguo | 8 | 9 | Ryan, CM | 66 |  |
| 10 | Wang Yan | 8 | 10 | Brands, AMA | 65 |  |
